# Peptide Linked Diacetylene Amphiphiles for Detection of Epitope Specific Antibodies

**DOI:** 10.3390/chemosensors10020062

**Published:** 2022-02-03

**Authors:** Natalie Tran, Priyanka Shiveshwarkar, Justyn Jaworski

**Affiliations:** Department of Bioengineering, University of Texas at Arlington, Arlington, TX 76010, USA

**Keywords:** peptide, amphiphile, epitope, antibody, detection

## Abstract

Antibodies produced in response to adaptive immunity provide a receptor with multiple sites for binding to a distinct epitope of an antigen. Determining antibody levels to specific antigens has important clinical applications in assessing immune status or deficiency, monitoring infectious or autoimmune diseases, and diagnosing allergies. Leveraging that a specific antibody will bind to a distinct small peptide epitope without requiring the entire antigen to be present, we demonstrate in this work a proof-of-concept assay to detect the presence of an antibody by using peptide epitopes linked to an amphiphile to generate a vesicle-based sensing system. By affording multiple copies of the epitope site on the vesicle, we revealed that the vesicles visibly aggregate in response to an antibody specific for that epitope due to multivalent binding provided by the antibody. We also uncovered the role of peptide surface density in providing accessible epitopes on the vesicles for antibody binding. In summary, using a peptide derived from the coat protein of human influenza virus directly linked to a diacetylene-containing amphiphile afforded peptide-laden vesicles that proved capable of detecting the presence of antibodies specific for human influenza hemagglutinin.

## 1. Introduction

The use of peptides as components of sensing systems is particularly attractive, as they are relatively small with straightforward means of synthesis as well as modification, and can provide high-affinity targets for receptor-specific interactions [1]. In addition, synthetic peptides can serve to mimic antigenic determinant sites within larger proteins of interest, and are thereby capable of binding with antibodies that have specificity toward those protein antigens [2–4]. Peptide-based sensors have been used to detect a variety of targets, including proteins, enzymes, and ions. Peptide-based sensing systems will generally exploit the molecular recognition capabilities between the peptide and a given target to serve as the selectivity element, with the coupling of the peptide to a transducer in order to enable signal detection by the user. Such transduction can be as simple as a change in fluorescence afforded by a pendent fluorophore [5], or more complex transduction, such as having the peptide coupled to a quartz crystal microbalance [6]. The ability to identify target-selective peptides to serve as recognition moieties has been demonstrated by evolutionary screening approaches [7], which opens the way for novel peptide-based biosensors [8,9]. The rational design of peptides can also be implemented for integration with transduction systems. For example, in the detection of Cu^2+^ ions using a fluorescein isothiocyanate-coupled peptide (FITC-Ahx-His-Glu-Phe-Cys-NH_2_), researchers have demonstrated that electron transfer occurs resulting in fluorescence quenching after Cu^2+^ binds with the histidine and cysteine, and which proceeds with high sensitivity [10]. Electrochemical sensing has also been demonstrated by designing peptide coatings on gold electrodes that serve as selective substrates for proteases [11]. In one manifestation, this has been used for the determination of metastasis-linked protease [12].

Peptides have also been proven to work well as responsive or selective moieties when linked to nanoparticles. Using Au nanoparticles functionalized with the peptide H_2_N-Leu-Aib-Tyr-OMe, pH-sensitive reversible aggregation has been demonstrated result in a visible red to violet color change [13]. This same peptide Au nanoparticle system was also found to be responsive to dosages of Hg^2+^ ions above 4 ppm [14], while other peptides functionalized to Au nanoparticles have demonstrated detection of other metal ions of interest [15–17]. In other manifestations, the coating of the receptive peptides to Au or ZnO nanoparticles enabled their high surface area display, enabling their use as a coating deposited onto quartz crystal microbalances for the sensing of different gases, including volatile alcohols and esters [18,19]. Peptides displayed on polymer nanoparticles in the form of polydiacetylene vesicles have also been implemented for detecting the presence of trinitrotoluene at relatively high concentrations [20], while utilizing the same vesicles for the coating of carbon nanotube-based sensors facilitated orders of magnitude increases in sensitivity with retained selectivity for trinitrotoluene [8]. Polydiacetylene vesicles provide a class of responsive materials that are particularly fitting for sensing applications due to their ability to provide optical responses in the form of changes in their florescence or absorbance in response to given stimuli depending on their structure [21]. Functionalizing the surface of polydiacetylene vesicles with receptive moieties thus provides a well-known approach for customizing their chromic and fluorogenic responses to targets [22]; however, an alternative mode of detection also exists, by which the colloidal stability of polydiacetylene vesicles can be modulated by the presence of target analytes, resulting in aggregation [23]. Using this aggregation strategy, the pendant side-chains of polydiacetylene vesicles have previously been decorated with chemical groups, including cyanuric acid for the detection of melamine [24], or oxime modifications to facilitate the detection of organophosphates [25]. In addition, we have recently shown that the peptides displayed on polydiacetylene vesicles that mimic biotin could facilitate the detection of streptavidin through multi-vesicle binding, which results in aggregation [26].

**Figure 1.**
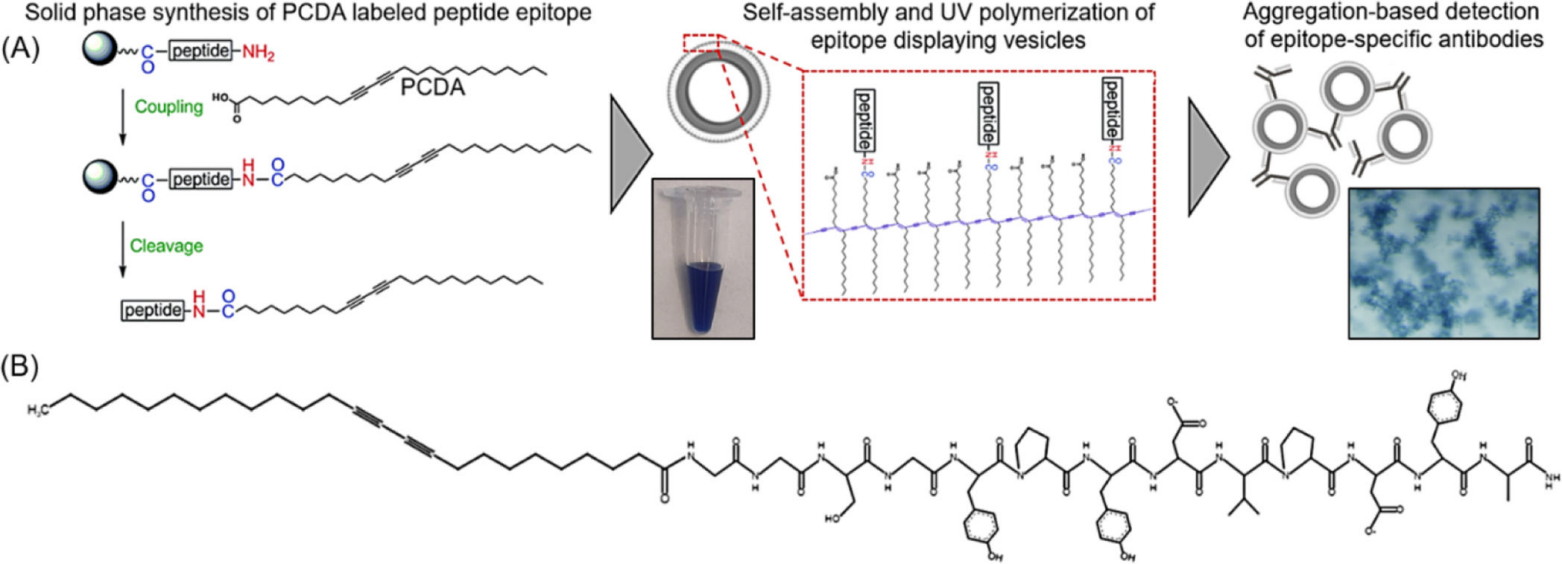
(**A**) Schematic overview of the process implemented in this work for generating a vesicle-based sensor with displayed hemagglutinin (HA) peptide epitope for the detection of epitope-specific anti-HA antibodies. (**B**) Sketch of the synthesized HA peptide epitope appended to 10,12 pentacosadiynoic acid (PCDA) via a Gly-Gly-Ser-Gly spacer segment.

In the following work, we extend this strategy to demonstrate that polydiacetylene-based vesicles displaying peptide epitopes can facilitate a straightforward aggregation test for the detection of specific antibodies, as seen in Figure 1. Immunoassays are widely used in biology and medicine for identifying antibodies in samples with common assays, including latex agglutination tests, lateral flow assays, and Luminex bead-based assays. Latex agglutination tests, for example, have been used to reveal the presence of antibodies within samples by the clumping or aggregation of antigen-coated latex beads, and depending on the format of the assay, have been used for determining immunity to viruses including SARS-CoV-2 [27–30], as well as detection of infection by bacteria or parasites [31,32]. Applying this principle, we reveal a strategy for the generation of epitope displaying polydiacetylene vesicles capable of being used in the visual detection of the presence of specific antibodies by target-specific binding to induce aggregation. In this proof-of-concept, we utilized the influenza hemagglutinin (HA) peptide epitope [33] (Tyr-Pro-Tyr-Asp-Val-Pro-Asp-Tyr-Ala) appended to an amphiphile of 10,12 pentacosadiynoic acid (PCDA) via a Gly-Gly-Ser-Gly spacer for the formation of epitope-decorated vesicles that facilitate the detection of the presence of the anti-HA antibody by aggregation. As there is not yet a single test capable of measuring the presence of each different antigen-specific antibody within a sample, separate antigen-labeled probes must be developed and implemented. The following work describes a streamlined synthetic approach for the rapid development of peptide epitope-labeled vesicles to serve as such antigen-labeled probes, which will facilitate aggregation-based assays in detecting the presence of specific antibodies. We believe this strategy will serve as a foundation for future work by developing a high-throughput approach for simultaneously detecting the presence of multiple distinct antigen-specific antibodies when loading the vesicles with unique reporter components.

## 2. Materials and Methods

### 2.1. Materials and Vesicle Fabrication

The synthesis of hemagglutinin (HA) epitope-labeled amphiphiles was carried out using the solid-phase peptide synthesis of the HA epitope sequence (Tyr-Pro-Tyr-Asp-Val-Pro-Asp-Tyr-Ala) with an N terminal Gly-Gly-Ser-Gly spacer prior to the final coupling reaction of 10,12 pentacosadiynoic acid (PCDA) to provide the diacetylene-containing amphiphile (Figure 1). A second control amphiphile in which the HA epitope was scrambled to have the sequence Tyr-Pro-Tyr-Asp-Pro-Ala-Asp-Val-Tyr with a Gly-Gly-Ser-Gly spacer followed by PCDA was also synthesized. Fmoc-protected amino acids were used for the solid phase synthesis of the peptides on a rink amide resin with HOBT/DIC coupling of the amino acids, deprotection of the Fmoc groups with piperidine, and Kaiser tests for confirmation at each step. After the final deprotection step, the HOBT/DIC coupling reaction was again used to cap PCDA to the free N terminus of the peptide. The samples were cleaved with TFA and precipitated in cold ether overnight at −20 °C. The product was then dried and analyzed by liquid chromatography mass spectrometry (LCMS) (Figure S1).

**Figure 2.**
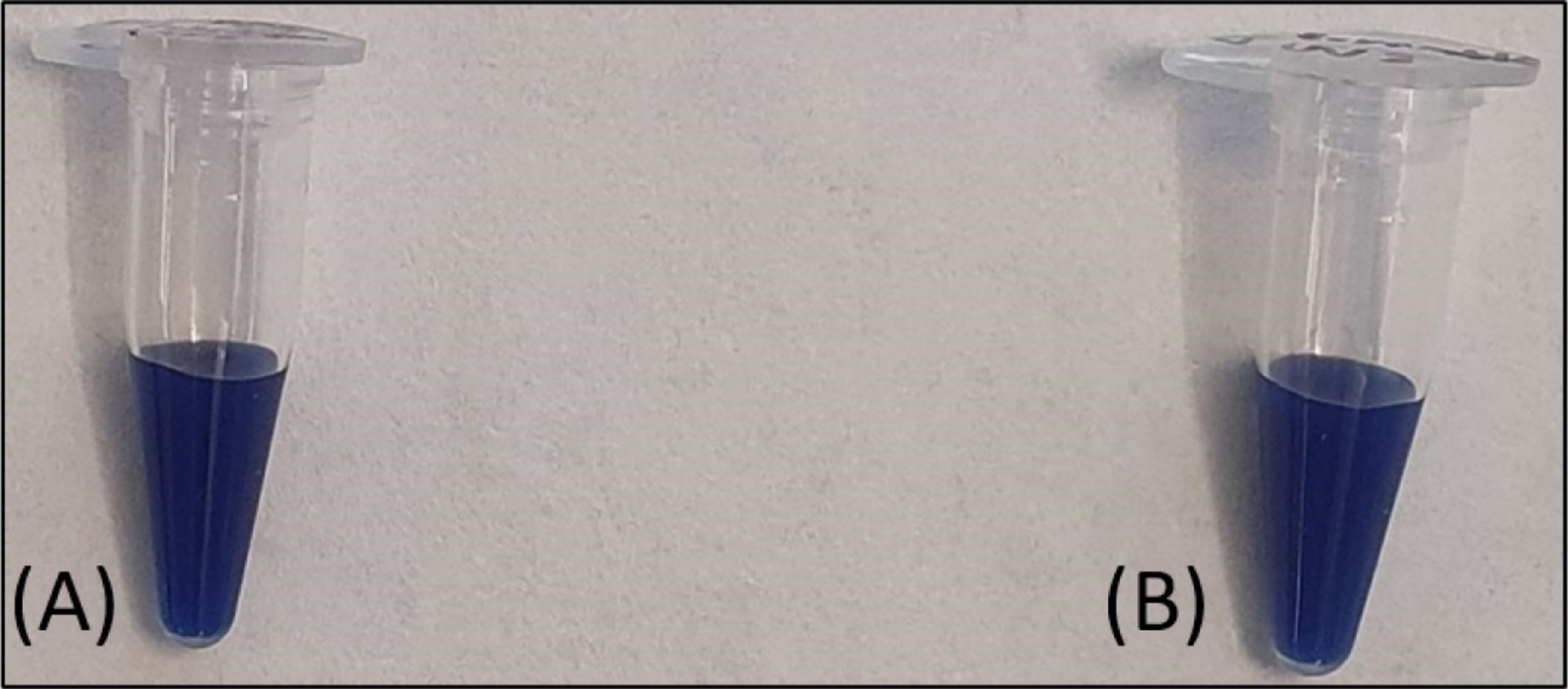
Suspensions of PCDA vesicles with (**A**) 0.01% HA epitope or (**B**) 0.01% scrambled HA epitope after 3 min of UV irradiation at 254 nm.

For vesicle formation, 50 umole of unlabeled PCDA amphiphile, 50 umole of HA epitope-labeled amphiphile, 50 umole of scrambled HA epitope-labeled amphiphile, and 50 umole of PEG3400-bis-PCDA (synthesized as described previously [34]) were dissolved separately in 200 uL of DMSO. Volumetrically, mixtures of the HA epitope-labeled amphiphile or the scrambled HA epitope-labeled amphiphile were combined with the unlabeled PCDA amphiphile and PEG3400-bis-PCDA to yield a total 50 umole of amphiphile, with different mole ratios of HA epitope-labeled amphiphile to unlabeled PCDA amphiphile and a constant 0.01% of PEG3400-bis-PCDA. Alternatively, for control vesicles, the 0.01% scrambled HA epitope-labeled amphiphile was combined with 0.01% PEG3400-bis-PCDA and 99.98% unlabeled PCDA amphiphile. The percentages of HA epitope-labeled PCDA in the mixtures included 0%, 0.01%, 0.1%, 1%, and 5% HA epitope-labeled PCDA. Vesicles were then formed by injecting the 50 umole mixtures into 7 mL of pre-heated (60 °C) 10 mM HEPES. The samples were continuously heated for 30 min, probe-sonicated for 30 min, and filtered with a 0.2 um syringe filter while hot. Vesicle self-assembly proceeded by incubating the samples at 4 °C overnight, and polymerization of the vesicles was carried out by UV irradiation (3–15 min at 254 nm). Vesicle stocks were stored at 4 °C, and an example of the product is shown in Figure 2.

### 2.2. HA Peptide Vesicle Assays with Anti-HA Beads

Anti-HA-coated magnetic beads (1, 5, or 10 uL) were used for the comparison of vesicle clearance by binding between the HA epitope presented on the vesicle and the anti-HA beads. We also examined the abilities of the vesicles with different mole percentages of the HA peptide epitope to be capture by the anti-HA antibody presented by the beads. The given amount of anti-HA bead stock was suspended in 50 uL of 10 mM HEPES containing 1% BSA and incubated at room temperature for 30 min with inversion of the tube every 5 min. The beads were collected by magnet followed by removal of the supernatant and resuspension in a vesicle solution containing 25 uL of 10 mM HEPES, 1%BSA, and 5 uL of PCDA vesicles displaying HA epitope ranging from 0 to 5%, as indicated. The samples were then incubated for 30 min with inversion as described above, and the anti-HA beads were collected by magnet. The supernatant was collected and analyzed by spectrophotometer, and the absorbance of the resulting supernatant was compared to that of the original vesicle suspension to assess the extent of vesicle clearance by the anti-HA beads. This assay was utilized to determine which HA epitope concentration displayed by the vesicles would provide superior anti-HA antibody binding, and that formulation was to be used for subsequent testing.

### 2.3. Vesicle–Antibody Aggregation Test

For aggregation testing with soluble antibody rather than bead-immobilized antibody, the following vesicle formulations were examined: 0% HA epitope vesicles, 0.01% HA epitope vesicles, and 0.01% scrambled HA epitope vesicles. The use of 0.01% HA epitope was selected based on its superior binding in the anti-HA bead assay described above. A mixture of 5 uL of UV polymerized vesicles was added to 20 uL of 10 mM HEPES, and the suspension was then mixed with 5 uL of unconjugated anti-HA antibody (dilutions of 1 mg/mL anti-HA antibody stock in 10 mM HEPES to different concentrations as indicated in the respective figures) and incubated at room temperature for 30 min. The samples were centrifuged at 5000 rpm for 5 min to expedite the precipitation of aggregates. Supernatant was collected and analyzed using an Epoch2 microplate spectrophotometer (BioTek Inc., Winooski, VT, USA) with Take3 plate attachment, and the absorbance of the resulting supernatant compared to that of the original vesicle suspension was used to determine the extent of antibody-induced aggregation relative to control samples. In addition, vesicles with either 0.01% HA epitope vesicles or 0.01% scrambled HA peptide were examined for anti-HA antibody concentration-dependent aggregation.

### 2.4. Anti-HA Bead Preparation

Anti-HA beads were prepared by non-covalently labeling protein A/G beads with anti-HA epitope antibody. In brief, 5 uL of protein A/G beads was suspended in 150 uL of 10 mM HEPES and the beads were then collected by magnet. The beads were then resuspended in 150 uL of HEPES containing 2 uL of anti-HA antibody. The sample was inverted once every 5 min for a total of 30 min followed by the collection of the beads and resuspension in HEPES containing 1% BSA. The beads were blocked with BSA for 30 min at room temperature with inversion of the tube every 5 min. The beads were collected and resuspended in fresh 1% BSA for immediate use in exposure to the vesicles for binding studies.

### 2.5. Microscale Thermophoresis

Additional examination of the 0.01% HA epitope vesicles binding to anti-HA antibody was carried out by microscale thermophoresis (MST). Vesicles with 0.01% HA epitope were heated at 75 °C for one minute to induce the characteristic red fluorescence of polydiacetylene to enable use of the vesicles as the reporter for the MST assay. Anti-HA antibody was diluted in 10 mM HEPES and added to the vesicles in equivalent volumes to provide a final antibody concentration of 250 ng/uL, 120 ng/uL, 60 ng/uL, 20 ng/uL, 6.9 ng/uL, or 0.63 ng/uL. The samples were immediately loaded into standard capillaries and allowed to incubate for an hour to allow adequate time for binding and aggregation. The samples were then loaded into a Nanotemper Monolith (NanoTemper Technologies, Inc., South San Francisco, CA, USA) and the MST traces were recorded using the provided MO.Control software.

## 3. Results

### 3.1. Comparing the Anti-HA Antibody Binding Ability of Vesicles with Different Percentages of HA Epitope

**Figure 3.**
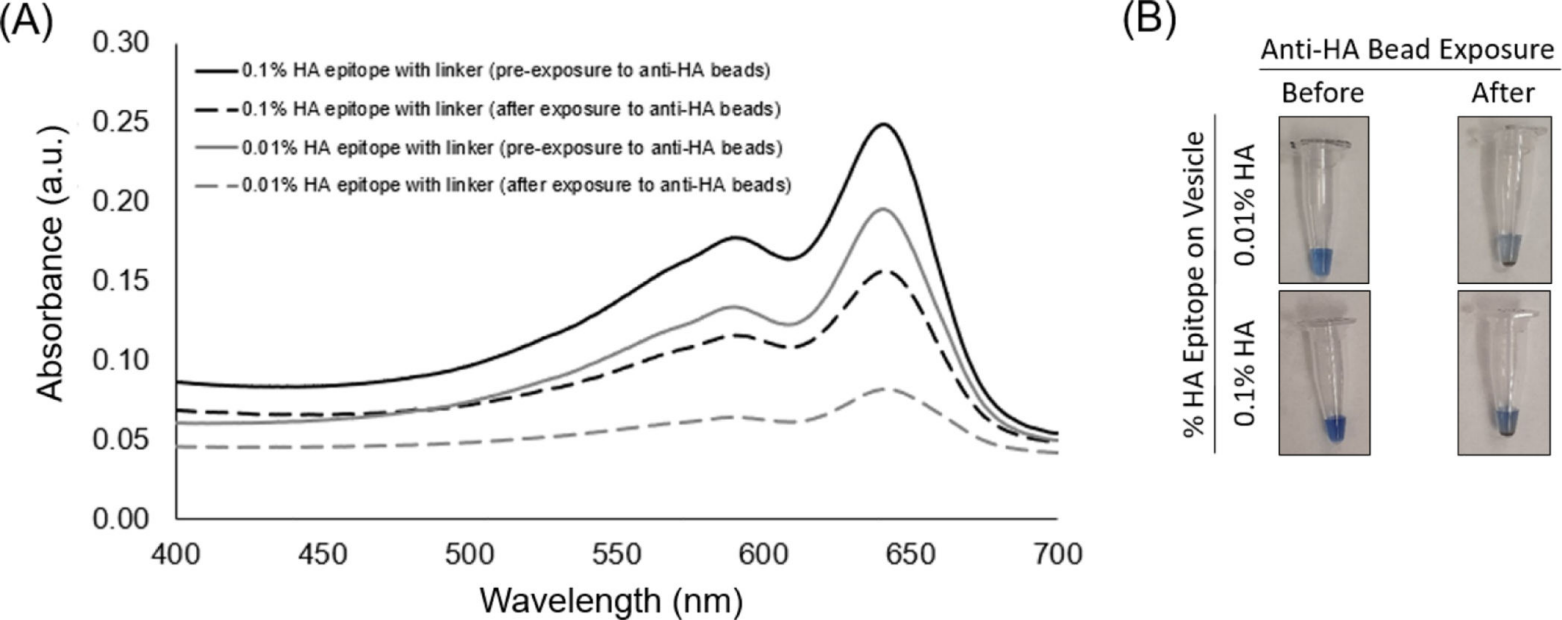
(**A**) Spectra and (**B**) images comparing vesicles with 0.1% or 0.01% HA epitope-labeled amphiphiles in terms of their ability to be bound and captured by anti-HA antibodies linked to magnetic beads.

The spectra of the UV-polymerized vesicles revealed a characteristic blue-phase polydiacetylene absorption maximum near ~645 nm for the different vesicle formulations containing 5%, 1%, 0.1%, 0.01%, or 0% HA peptide epitope. The exposure of the vesicles to anti-HA antibody-coated magnetic beads was used to assess the ability of the vesicles to bind with the anti-HA antibody as a function of the percent of HA epitope displayed by the vesicles. When vesicles bind to the anti-HA antibody, they can be pulled down by applying a magnet to provide clearance of the vesicles, as seen by the decrease in the intensity of the absorbance spectra. In Figure 3, we specifically show the absorbance spectra of the solution prior to and after exposure to the anti-HA beads for the case of vesicles with either 0.1% or 0.01% HA epitope. An important observation is that vesicles with 0.01% HA epitope exhibited a greater extent of clearance (reduction in the absorbance spectra) by the anti-HA beads as compared to vesicles with 0.1% HA epitope. Since the absorbance peaks correspond to the amount of polymerized vesicles within the suspension, this indicates that the 0.01% HA epitope vesicles are binding to, and thus being captured by, the anti-HA beads to a greater extent than the 0.1% HA epitope vesicles. From Figure S4, we can see this trend continues, showing even lower rates of the capture of vesicles for those having a higher percentage of epitopes (1% and 5%), which may be attributed to the steric hindrance of anti-HA antibody binding resulting from the overcrowding of epitopes on the vesicle surface.

**Figure 4.**
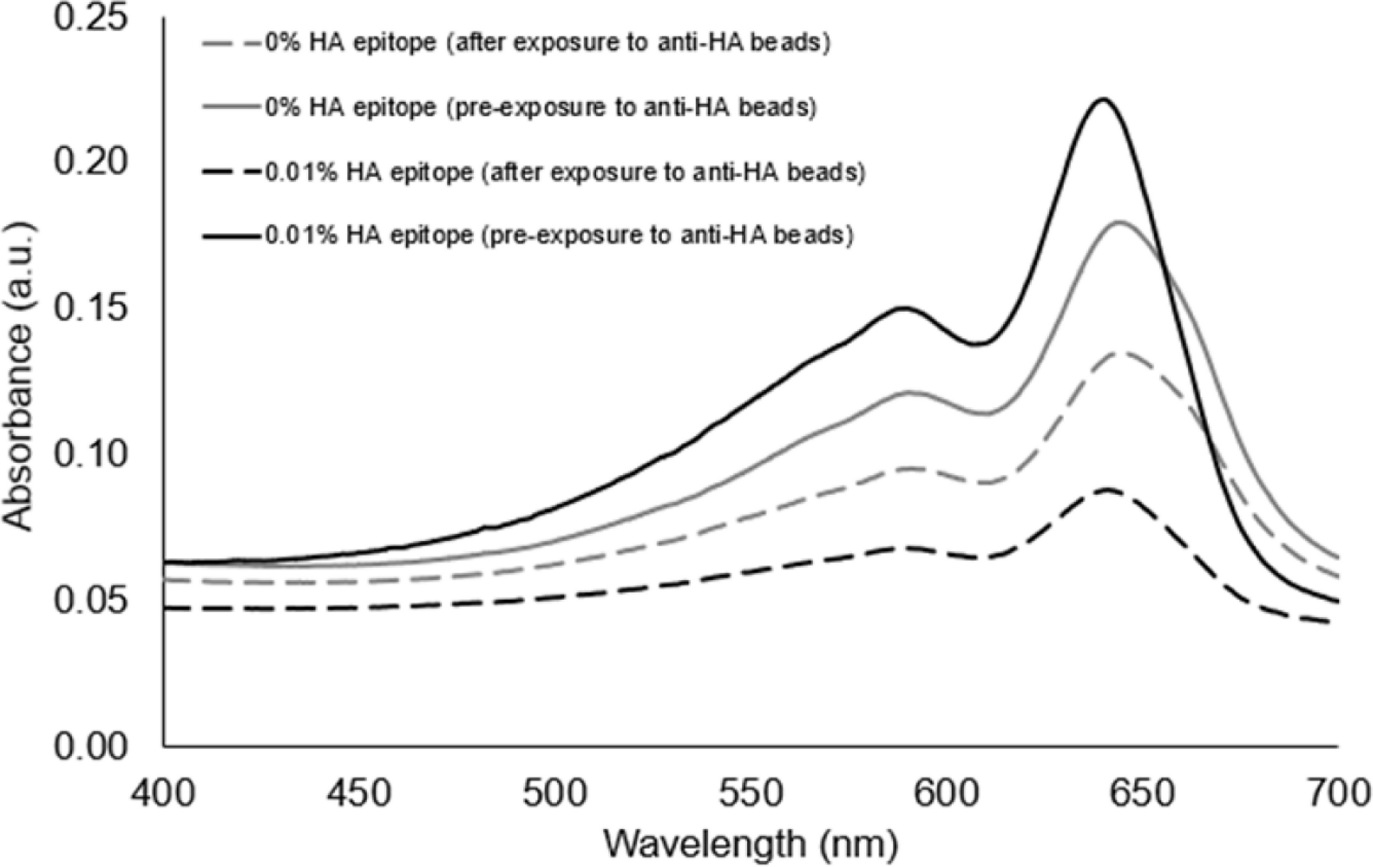
Comparison of vesicles with 0% or 0.01% HA epitope-labeled amphiphiles in terms of their ability to be bound and captured by anti-HA antibodies linked to magnetic beads.

To confirm that the clearance of vesicles from the suspension was due to the presence of the HA epitope binding to the anti-HA beads, we compared PCDA vesicles with no HA epitope to vesicles with 0.01% HA epitope amphiphile. As seen in Figure 4, the presence of the HA epitope on the vesicles facilitated a larger extent of vesicle clearance by the anti-HA beads as compared to the vesicles with 0% HA epitope.

### 3.2. Detecting the Presence of Soluble Anti-HA Antibody

**Figure 5.**
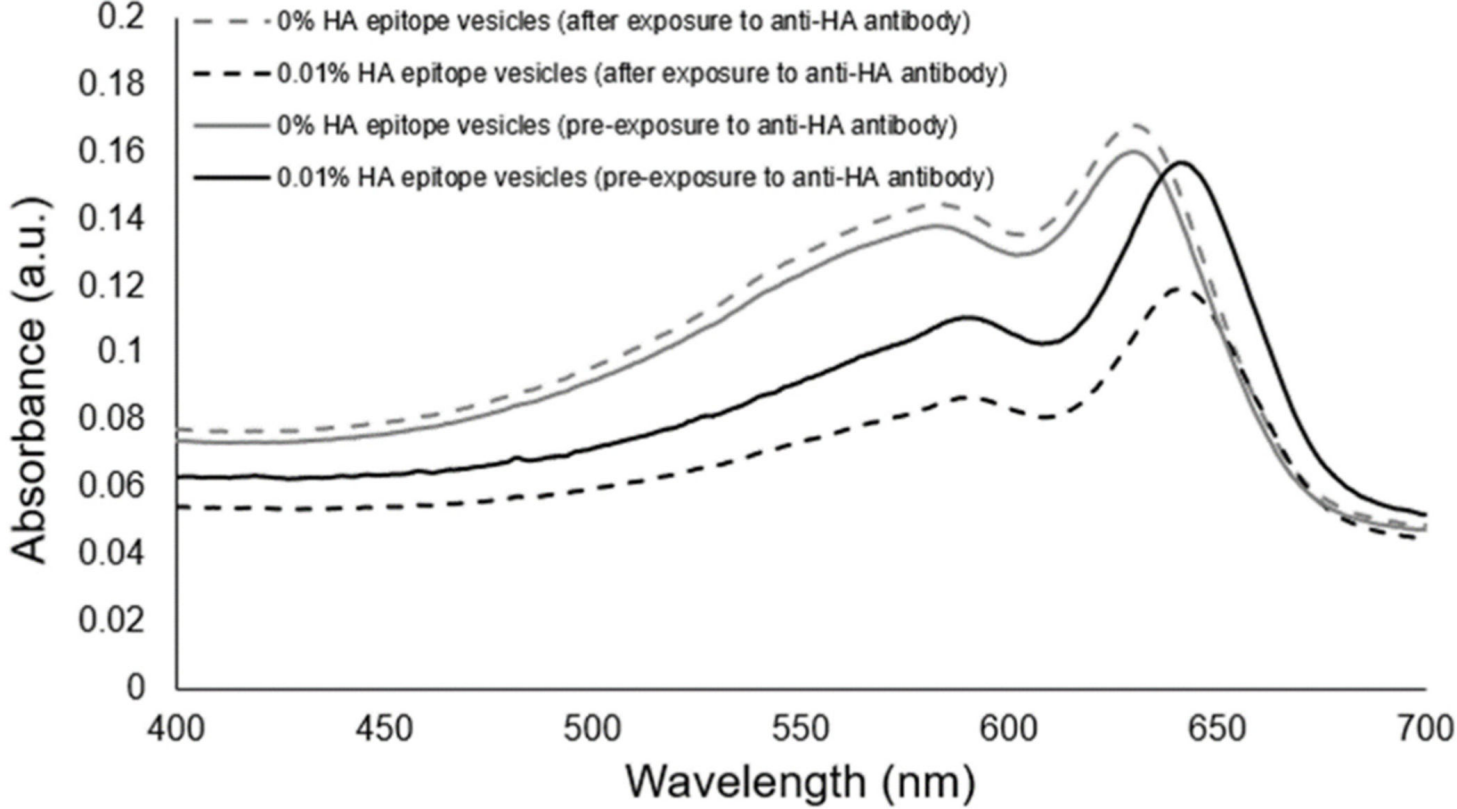
Examination of aggregation response to the presence of soluble anti-HA antibody for 0.01% HA epitope-displaying vesicles compared to the no-HA epitope control vesicles.

To examine the aggregation response of our HA epitope-displaying vesicles as a standalone sensor, we introduced soluble anti-HA antibody to vesicles possessing 0.01% HA epitope, and to serve as a comparison, we also provided anti-HA antibody to vesicles possessing 0% HA epitope. Figure 5 shows that the absorbance spectra revealed no major differences from before to after the exposure of the control vesicles to the anti-HA antibody. In contrast, the aggregation of the 0.01% HA epitope vesicles due to the presence of anti-HA antibody resulted in an observable aggregation providing a substantial reduction in the absorbance spectra.

**Figure 6.**
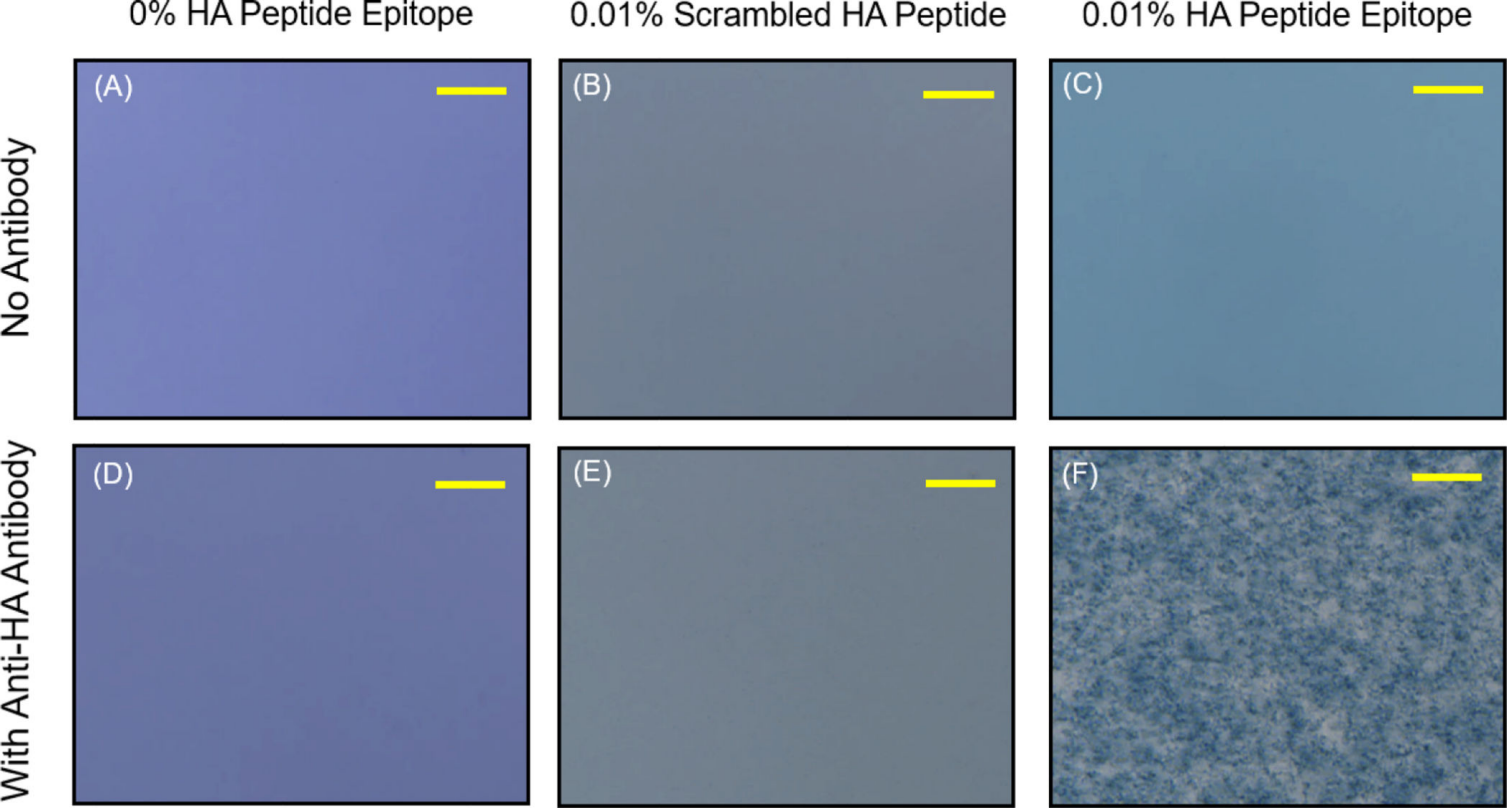
Phase contrast microscopy (scale bar = 10 um) images of vesicle samples displaying 0% HA epitope, 0.01% scrambled HA peptide, or 0.01% HA peptide epitope (**A**–**C**) without the addition of antibody as compared to those samples (**D**–**F**) with the addition of soluble anti-HA antibody (33 ng/uL), revealing aggregation specifically for the case of those vesicles possessing 0.01% HA peptide epitope.

In addition, we confirmed that the aggregation of the 0.01% HA epitope vesicles was the result of specific binding between the HA peptide epitope and the anti-HA antibody by comparing our results to those for vesicles possessing 0.01% scrambled HA peptide sequence, which would not be specifically recognized by the antibody. As seen below in Figure 6, the vesicle suspensions without the addition of anti-HA antibody appeared stable; however, the addition of the anti-HA antibody caused aggregation only for the vesicles bearing the HA peptide epitope. No visual aggregation could be seen by phase contrast microscopy for the vesicles displaying the scrambled HA peptide, or those displaying no peptide at all. This specific aggregation serves as the means for the transduction of the specific binding of antibodies toward those epitopes displayed by the vesicles into a visually detectable signal.

**Figure 7.**
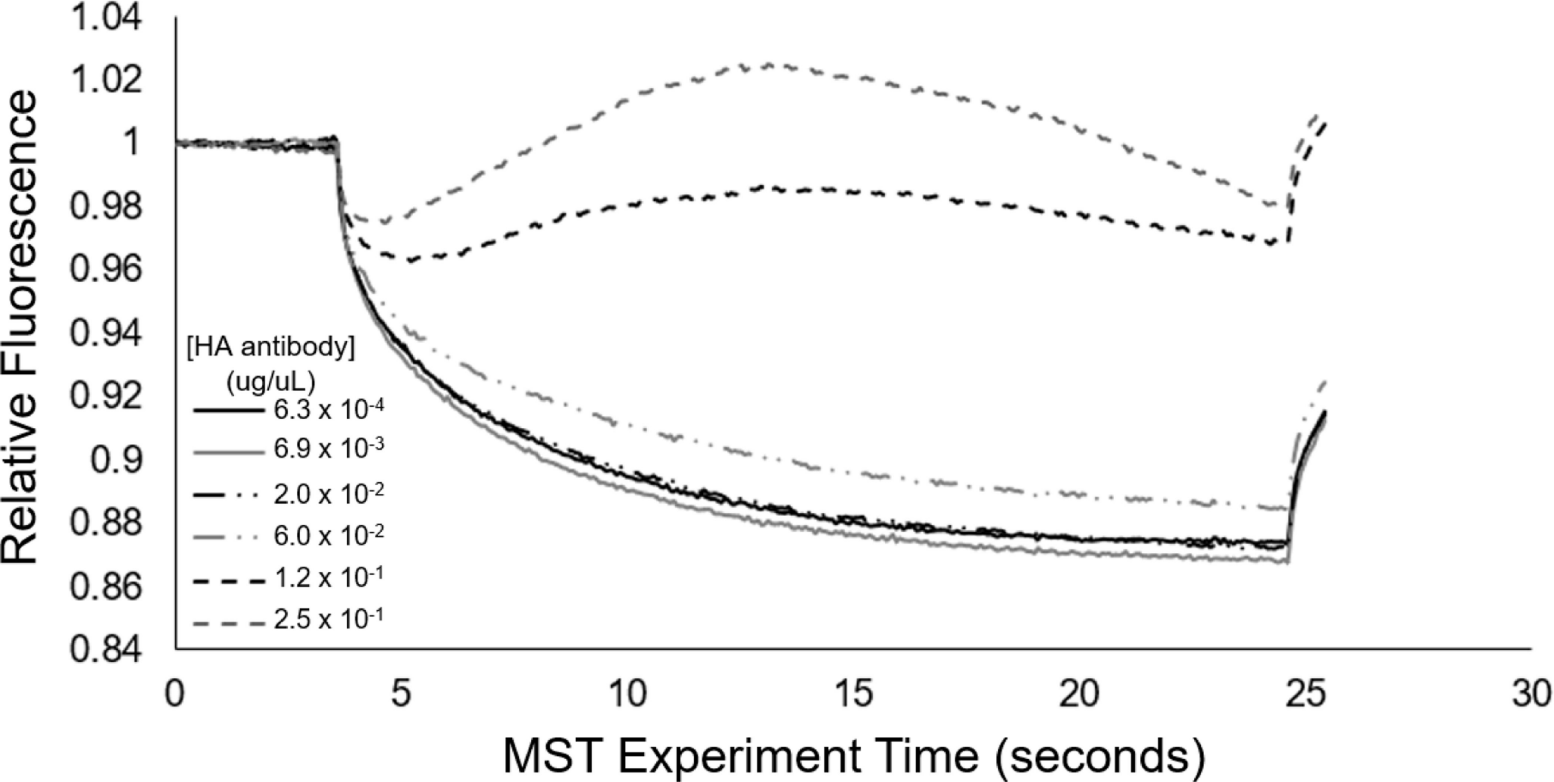
Microscale thermophoresis of 0.01% HA epitope-displaying vesicles (pre-heated to exhibit red fluorescence) mixed with concentrations of anti-HA antibody from 250 ng/uL to 0.63 ng/uL.

Microscale thermophoresis (MST) provides a sensitive means to assess changes in particle motion due to binding, and thus was employed as an additional approach to examine the concentration-dependent interactions between the anti-HA antibody and the 0.01% HA epitope-displaying vesicles. In Figure 7, at higher concentrations of the anti-HA antibody, we see the occurrence of positive thermophoresis in the 0.01% HA epitope vesicles, and this observed effect occurs with the increasing concentration of the target antibody, indicative of binding. Irregularities in the MST traces that suggest the formation of aggregates were also observed. Based on MST, the 0.01% HA epitope vesicles exhibited this aggregation behavior at 60 ng/uL of anti-HA antibodies and above; however, at anti-HA antibody concentrations of 20 ng/uL and below, this aggregation behavior was not observed.

When further examining the concentration-dependent aggregation response using visible spectrophotometer, the aggregation behavior for the 0.01% HA epitope vesicles revealed high aggregation, as shown by the attenuation in the spectra at an antibody concentration of 33 ng/uL and above. Concentrations lower than 33 ng/uL did not result in as noticeable a reduction in the spectra relative to the no-antibody control, which would suggest that the detection limit for the 0.01% HA epitope vesicles is near 33 ng/uL of anti-HA antibody under these conditions. In comparison, the vesicles with 0.01% scrambled HA peptide did not demonstrate a large attenuation in the spectra relative to the no-antibody control, and also did not reveal an anti-HA antibody-dependent decrease in the absorbance spectra.

## 4. Discussion

The aim of this study was to examine conditions for using epitope-labeled vesicles for detecting a response to the presence of epitope-specific antibodies in the form of aggregation behavior, which could be observed visually as well as by spectral readout. In this work, for testing purposes, we specifically used the common influenza hemagglutinin (HA) peptide epitope to display on our vesicles via a spacer and with different surface densities in order to examine the aggregation response to anti-HA antibody. As shown in Figure 3, the UV-polymerized vesicles exhibited their characteristic absorption maxima around 645 nm, which is known to result from the overlapping p orbitals within the conjugated diacetylene backbone. Using this absorption peak, we could compare the amount of vesicle aggregation by the loss of color represented as an attenuation in the absorbance spectra. Our first assessment of the HA epitope-displaying vesicles was performed via their ability to bind with anti-HA antibodies by directly examining the spectra intensity of the vesicle solution before and after exposure to anti-HA antibody-coated magnetic beads. The extent of capture of HA epitope-displaying vesicles by anti-HA beads was found to be impacted by the surface density of the HA epitope amphiphile on the vesicles, with a more substantial attenuation in the absorption spectra being observed when using 0.01% HA epitope vesicles as compared to 0.1% HA epitope vesicles. When compared to higher concentrations of 1% and 5% HA epitope (Figure S4), we observed even less capture by the anti-HA beads, indicating the surface density of epitopes as a critical factor that can be optimized. This trend indicates that the lower percentage of HA epitope displayed on the vesicles provides for better attenuation in signal due to binding and capturing by the anti-HA beads, while the 0% HA epitope vesicles (having no HA epitope) exhibited only marginal attenuation in signal, as seen in Figure 4. It may be deduced, then, that a high epitope density may cause some form of steric hindrance in which neighboring moieties may block the access of the antibody to the epitope. This is reasonable, given that, in prior works, we have found that as the surface densities of the displayed epitopes on PCDA vesicles increase, there is a point at which vesicle formation becomes hindered, resulting in the inadequate packing of the amphiphiles. Having multiple copies of the epitope site displayed by the vesicles is important in allowing the vesicles to bind with multiple antibodies simultaneously in order to facilitate a mechanism for vesicle aggregation, but displaying too many copies of the epitope can sterically restrict their access to antibodies.

Having confirmed the ability of the HA epitope-displaying vesicles to bind with the anti-HA antibody presented on beads, we then used the 0.01% HA epitope vesicles to test whether they could facilitate the aggregation-based detection of the presence of soluble anti-HA antibody. Figure 5 reveals that the 0.01% HA-displaying vesicles were able to aggregate within 30 min of exposure to the anti-HA antibody, and that an observable attenuation in the spectra resulted, while no aggregation or attenuation was observed in the spectra of the control PCDA vesicles having no HA epitope.

To examine the specificity of the observed vesicle aggregation, we produced another vesicle formulation displaying a scrambled HA epitope peptide sequence that will not bind specifically to the anti-HA antibody’s complementarity determining regions. As shown in Figure 6, the formation of aggregates of 0.01% HA epitope vesicles due to specific interactions between the anti-HA antibody and the displayed HA peptide epitope was confirmed, given that the 0.01% scrambled HA peptide did not produce aggregates when the anti-HA antibody was added. The specificity of vesicle aggregation as determined by the peptide epitopes displayed by the vesicles and the presence of corresponding anti-epitope antibodies thereby establishes this approach as a means for detecting the presence of specific antibodies. The antibody-induced aggregation signal could be observed at as low as 33 ng/uL under the current conditions and vesicle configuration.

We have demonstrated that this approach provides for binary sensing in determining if the specific anti-HA antibody is present above a specified concentration. In doing so, this technique may find a use similar to latex agglutination assays to provide semi-quantitative analysis of antibody titers. In binary sensing approaches like latex agglutination tests, the antibody concentration can be determined by setting up a series of dilutions followed by the assessment of aggregation to identify the greatest dilution factor that will still provide a positive aggregation result, which corresponds to the titer. Our vesicles may similarly find use in such a semi-quantitative approach. The use of binary testing alone can have a number of other applications in areas where knowledge of a positive or negative result rather than an exact antibody concentration is worthwhile.

**Figure 8.**
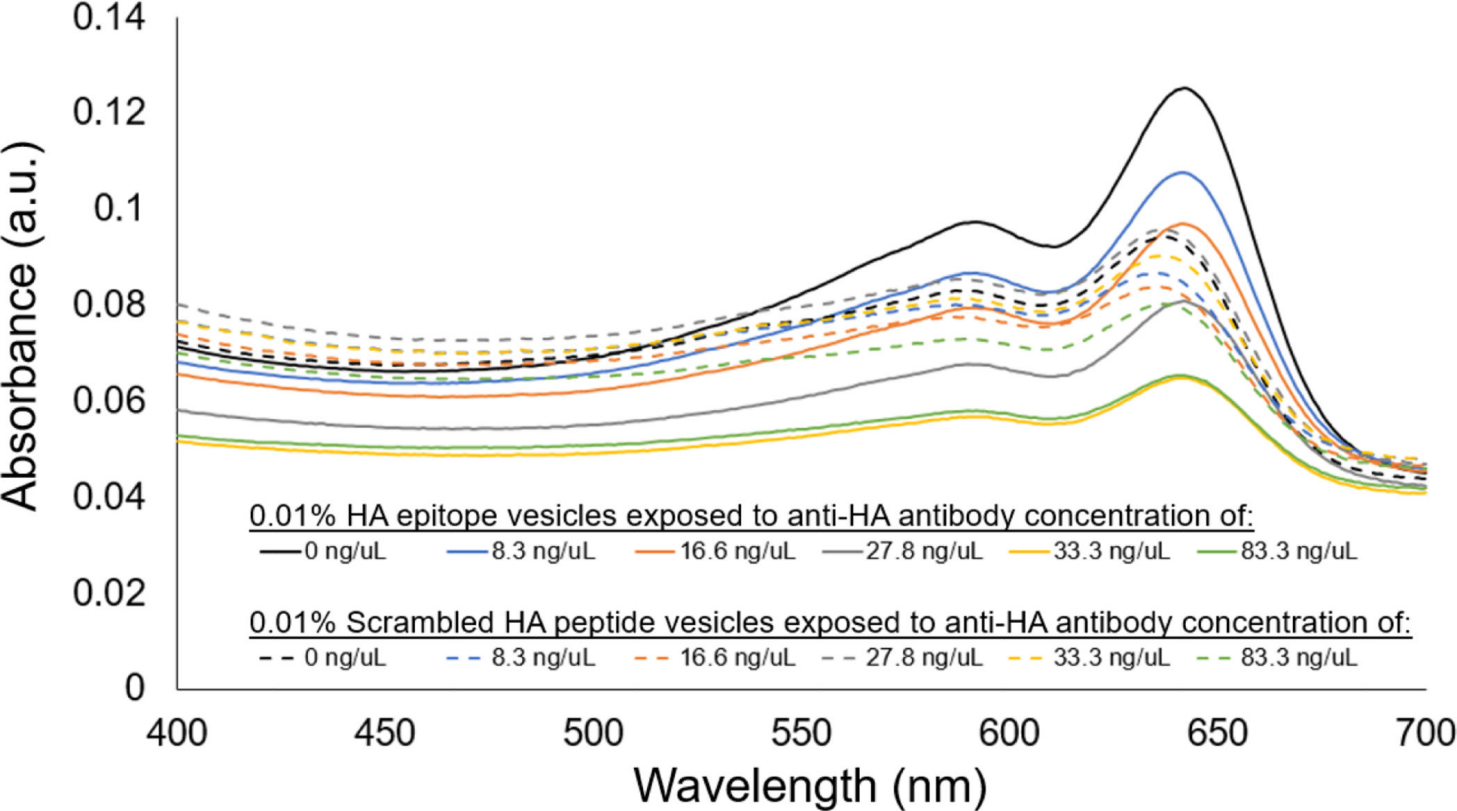
Visible spectra showing anti-HA antibody concentration-dependent aggregation response of 0.01% HA epitope-displaying vesicles (solid lines), with no concentration-dependent response observed for 0.01% scrambled HA peptide vesicles (dashed lines).

To serve as a mode of cross-validation for the aggregation response behavior and to quantitatively assess the transient aggregation response, we also implemented microscale thermophoresis (MST). From Figure 7, we see that the MST trace caused a concentration-dependent change in the thermophoretic mobility. Specifically, at high concentrations of antibody (>60 ng/uL), the MST traces revealed substantial increases in the levels of relative fluorescence, which are indicative of positive thermophoresis [35], and provided indication of 0.01% HA epitope vesicle aggregate formation due to anti-HA antibody binding, resulting in increased aggregate size and less thermophoretic movement. At anti-HA antibody concentrations of 20 ng/uL and below, the aggregate formation of the 0.01% HA epitope vesicles was not observed by MST. This result coincides with our spectral observations (Figure 8) of the aggregation behavior for the 0.01% HA epitope vesicles, which revealed high levels of aggregation at anti-HA antibody concentrations of 33 ng/uL and above, but less aggregation at lower concentrations.

In looking at the utility of the HA epitope-labeled vesicles for aggregation-based detection, we can see that the use of a soluble anti-HA antibody provides aggregation, but by centrifuging the samples, we can derive a more stark formation of the precipitate. While it is unlikely that centrifugation will be readily available in all contexts, the use of a simple magnetic bead assay may be more widely deployable. Thus, we conducted several optimization experiments to enhance the bead-based assay. Initially we examined the production of our own anti-HA beads by labeling the anti-HA antibody onto magnetic protein A/G beads. To assess whether the protein A/G beads were fully coated with anti-HA antibody, we examined the clearance of HA epitope-containing vesicles when exposed to protein A/G beads produced with increasing amounts of anti-HA antibody. Our data in Figure S2 suggest that we were able to provide a maximum (saturated) amount of antibody on the protein A/G beads, as only minimal increases in the binding of our 0.01% HA epitope vesicles were observed when using twice the saturating amount of antibody. If this were not the case, we would have expected the capture of a greater amount of the 0.01% HA epitope vesicles when using a higher amount of anti-HA antibody labeling on the beads. To assess whether we were utilizing the optimal amount of anti-HA beads, we also examined the extent of 0.01% HA epitope-labeled vesicle clearance as a function of the amount of anti-HA beads used. From Figure S3, it can be seen that utilizing a larger amount of anti-HA beads can facilitate a higher degree of clearance of the 0.01% HA epitope-displaying vesicles. This can dramatically improves the clarity of readout, and may be the best way forward in the future use of this vesicle-based sensing approach for the profiling of epitope-specific antibodies. To summarize, we have shown here our proof-of-concept epitope-labeled vesicles for detecting the presence of the epitope-specific antibody; specifically in this case, those antibodies against human influenza agglutinin. We look forward to future work examining the application areas of this form of aggregation-based sensor for antibody detection, and its ability to be customized for other epitopes by virtue of its modularity.

## Supplementary Material

Supplementary Information

## References

[1] Pazos E; Vazquez O; Mascarenas JL; Vazquez ME Peptide-based fluorescent biosensors. Chem. Soc. Rev 2009, 38, 3348–3359. 20449054 10.1039/b908546g

[2] Wilson IA; Niman HL; Houghten RA; Cherenson AR; Connolly ML; Lerner RA The structure of an antigenic determinant in a protein. Cell 1984, 37, 767–778. 6204768 10.1016/0092-8674(84)90412-4

[3] Houghten RA; Pinilla C; Blondelle SE; Appel JR; Dooley CT; Cuervo JH Generation and use of synthetic peptide combinatorial libraries for basic research and drug discovery. Nature 1991, 354, 84–86. 1719428 10.1038/354084a0

[4] Dyson HJ; Lerner RA; Wright PE The physical basis for induction of protein-reactive antipeptide antibodies. Annu. Rev. Biophys. Biophys. Chem 1988, 17, 305–324. 2456075 10.1146/annurev.bb.17.060188.001513

[5] Kwak E-A; Kydd L; Lim B; Jaworski J. IR-783 Labeling of a Peptide Receptor for ‘Turn-On’Fluorescence Based Sensing. Chemosensors 2018, 6, 47. 31080779 10.3390/chemosensors6040047 PMC6510487

[6] Wasilewski T; Szulczyński B; Wojciechowski M; Kamysz W; Gębicki J. Determination of long-chain aldehydes using a novel quartz crystal microbalance sensor based on a biomimetic peptide. Microchem. J 2020, 154, 104509.

[7] Jaworski JW; Raorane D; Huh JH; Majumdar A; Lee S-W Evolutionary screening of biomimetic coatings for selective detection of explosives. Langmuir 2008, 24, 4938–4943. 18363413 10.1021/la7035289

[8] Kim TH; Lee BY; Jaworski J; Yokoyama K; Chung W-J; Wang E; Hong S; Majumdar A; Lee S-W Selective and sensitive TNT sensors using biomimetic polydiacetylene-coated CNT-FETs. ACS Nano 2011, 5, 2824–2830. 21361351 10.1021/nn103324p

[9] Cerruti M; Jaworski J; Raorane D; Zueger C; Varadarajan J; Carraro C; Lee S-W; Maboudian R; Majumdar A. Polymer-oligopeptide composite coating for selective detection of explosives in water. Anal. Chem 2009, 81, 4192–4199. 19476386 10.1021/ac8019174

[10] Hao C; Guo X; Lai Q; Li Y; Fan B; Zeng G; He Z; Wu J. Peptide-based fluorescent chemical sensors for the specific detection of Cu^2+^ and S^2−^. Inorg. Chim. Acta 2020, 513, 119943.

[11] Wcisło A; Małuch I; Niedziałkowski P; Ossowski T; Prahl A. Label-free electrochemical test of protease interaction with a peptide substrate modified gold electrode. Chemosensors 2021, 9, 199.

[12] Muñoz-San Martín C; Pedrero M; Gamella M; Montero-Calle A; Barderas R; Campuzano S; Pingarrón JM A novel peptide-based electrochemical biosensor for the determination of a metastasis-linked protease in pancreatic cancer cells. Anal. Bioanal. Chem 2020, 412, 6177–6188. 31989193 10.1007/s00216-020-02418-w

[13] Si S; Mandal TK pH-controlled reversible assembly of peptide-functionalized gold nanoparticles. Langmuir 2007, 23, 190–195. 17190503 10.1021/la061505r

[14] Si S; Kotal A; Mandal TK One-dimensional assembly of peptide-functionalized gold nanoparticles: An approach toward mercury ion sensing. J. Phys. Chem. C 2007, 111, 1248–1255.

[15] Yang T; Zhang X-X; Yang J-Y; Wang Y-T; Chen M-L Screening arsenic (III)-binding peptide for colorimetric detection of arsenic (III) based on the peptide induced aggregation of gold nanoparticles. Talanta 2018, 177, 212–216. 29108578 10.1016/j.talanta.2017.07.005

[16] Zhu D; Li X; Liu X; Wang J; Wang Z. Designing bifunctionalized gold nanoparticle for colorimetric detection of Pb^2+^ under physiological condition. Biosens. Bioelectron 2012, 31, 505–509. 22138466 10.1016/j.bios.2011.11.026

[17] Zhang M; Liu Y-Q; Ye B-C Colorimetric assay for parallel detection of Cd^2+^, Ni^2+^ and Co^2+^ using peptide-modified gold nanoparticles. Analyst 2012, 137, 601–607. 22158918 10.1039/c1an15909g

[18] Mascini M; Gaggiotti S; Della Pelle F; Di Natale C; Qakala S; Iwuoha E; Pittia P; Compagnone D. Peptide modified ZnO nanoparticles as gas sensors array for volatile organic compounds (VOCs). Front. Chem 2018, 6, 105. 29713626 10.3389/fchem.2018.00105 PMC5911495

[19] Compagnone D; Fusella G; Del Carlo M; Pittia P; Martinelli E; Tortora L; Paolesse R; Di Natale C. Gold nanoparticles-peptide based gas sensor arrays for the detection of foodaromas. Biosens. Bioelectron 2013, 42, 618–625. 23261699 10.1016/j.bios.2012.10.096

[20] Jaworski J; Yokoyama K; Zueger C; Chung W-J; Lee S-W; Majumdar A. Polydiacetylene incorporated with peptide receptors for the detection of trinitrotoluene explosives. Langmuir 2011, 27, 3180–3187. 21275406 10.1021/la104476p

[21] Yarimaga O; Jaworski J; Yoon B; Kim J-M Polydiacetylenes: Supramolecular smart materials with a structural hierarchy for sensing, imaging and display applications. Chem. Commun 2012, 48, 2469–2485. 10.1039/c2cc17441c 22281683

[22] Reichert A; Nagy JO; Spevak W; Charych D. Polydiacetylene liposomes functionalized with sialic acid bind and colorimetrically detect influenza virus. J. Am. Chem. Soc 1995, 117, 829–830.

[23] Cho Y-S; Ahn KH Molecular interactions between charged macromolecules: Colorimetric detection and quantification of heparin with a polydiacetylene liposome. J. Mater. Chem. B 2013, 1, 1182–1189. 32260841 10.1039/c2tb00410k

[24] Lee J; Jeong EJ; Kim J. Selective and sensitive detection of melamine by intra/inter liposomal interaction of polydiacetylene liposomes. Chem. Commun 2011, 47, 358–360. 10.1039/c0cc02183k 20838687

[25] Lee J; Seo S; Kim J. Colorimetric Detection of Warfare Gases by Polydiacetylenes Toward Equipment-Free Detection. Adv. Funct. Mater 2012, 22, 1632–1638.

[26] Shrestha A; Lim B; Shiveshwarkar P; Rojas G; Abure I; Nelson AD; Jaworski J. Self-Assembled Peptide-Labeled Probes for Agglutination-Based Sensing. Macromol. Res 2021, 29, 577–581. 34955698 10.1007/s13233-021-9079-3 PMC8693994

[27] Gershon AA; LaRussa P; Steinberg S. Detection of antibodies to varicella-zoster virus using a latex agglutination assay. Clin. Diagn. Virol 1994, 2, 271–277. 15566772 10.1016/0928-0197(94)90051-5

[28] Voss C; Esmail S; Liu X; Knauer MJ; Ackloo S; Kaneko T; Lowes L; Stogios P; Seitova A; Hutchinson A. Epitope-resolved serology test differentiates the clinical outcome of COVID-19 and identifies defects in antibody response in SARS-CoV-2 variants. medRxiv 2021.

[29] Esmail S; Knauer MJ; Abdoh H; Voss C; Chin-Yee B; Stogios P; Seitova A; Hutchinson A; Yusifov F; Skarina T. Rapid and accurate agglutination-based testing for SARS-CoV-2 antibodies. Cell Rep. Methods 2021, 1, 100011. 10.1016/j.crmeth.2021.100011 PMC8114573 34235498

[30] Breuer J; Schmid DS; Gershon AA Use and limitations of varicella-zoster virus-specific serological testing to evaluate breakthrough disease in vaccinees and to screen for susceptibility to varicella. J. Infect. Dis 2008, 197, S147–S151. 18419389 10.1086/529448

[31] Polpanich D; Tangboriboonrat P; Elaissari A; Udomsangpetch R. Detection of malaria infection via latex agglutination assay. Anal. Chem 2007, 79, 4690–4695. 17511424 10.1021/ac070502w

[32] Mazumder P; Chuang H; Wentz MW; Wiedbrauk DL Latex agglutination test for detection of antibodies to Toxoplasma gondii. J. Clin. Microbiol 1988, 26, 2444–2446. 3235675 10.1128/jcm.26.11.2444-2446.1988 PMC266914

[33] Field J; Nikawa J.-l.; Broek D; MacDonald B; Rodgers L; Wilson I; Lerner R; Wigler M. Purification of a RAS-responsive adenylyl cyclase complex from Saccharomyces cerevisiae by use of an epitope addition method. Mol. Cell. Biol 1988, 8, 2159–2165. 2455217 10.1128/mcb.8.5.2159-2165.1988 PMC363397

[34] Rojas G; Shiveshwarkar P; Lim B; Shrestha A; Abure I; Nelson A; Jaworski J. Modifying Polydiacetylene Vesicle Compositions to Reduce Non-Specific Interactions. Macromol. Res 2021, 29, 449–452. 35321256 10.1007/s13233-021-9059-7 PMC8936729

[35] Rainard JM; Pandarakalam GC; McElroy SP Using microscale thermophoresis to characterize hits from high-throughput screening: A European lead factory perspective. SLAS Discov. Adv. Life Sci. RD 2018, 23, 225–241. 10.1177/2472555217744728 PMC5824829 29460707

