# Supplementary material for: Peptide Linked Diacetylene Amphiphiles for Detection of Epitope Specific Antibodies
Source: Chemosensors (Basel). Author manuscript; Available in PMC 2022 Dec 19. (PMC9762857; doi:10.3390/chemosensors10020062)
Supplement: Supplementary Information [file NIHMS1857516-supplement-Supplementary_Information.pdf]

Supplementary Data

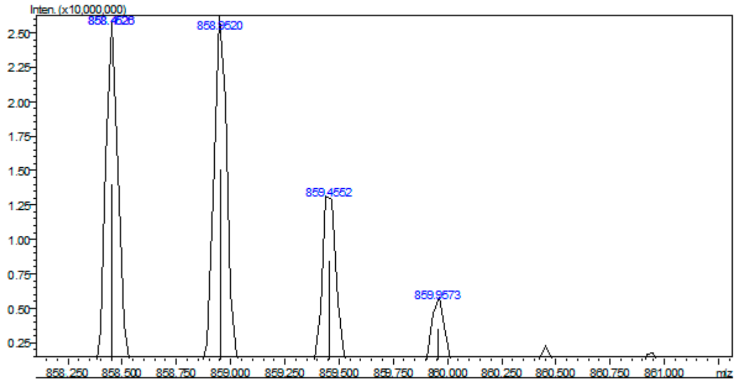

Figure S1. LCMS mass spectrum of synthesized PCDA-Gly-Gly-Ser-Gly-Tyr-Pro-Tyr-Asp-Val-Pro-Asp-Tyr-Ala amphiphile which bears the HA epitope. The calculated m/z for this product is 1716.98 (for single charge ion peak) or 858.49 (for double charged ion peak), where the double peak can be observed in the mass spectrum.

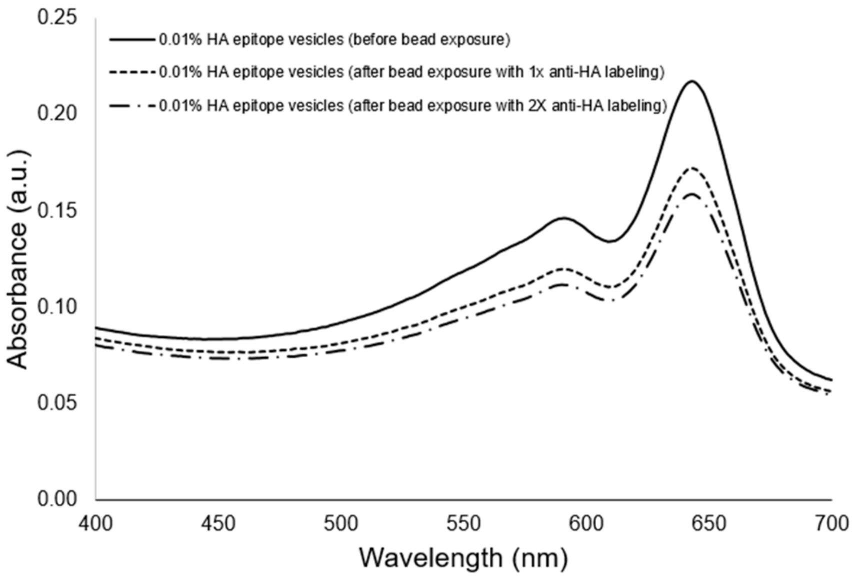

Figure S2. Absorption spectra of 0.01% HA epitope displaying vesicles before and after exposure to anti-HA beads produced from magnetic protein A/G beads non-covalently labeled with anti-HA antibody.

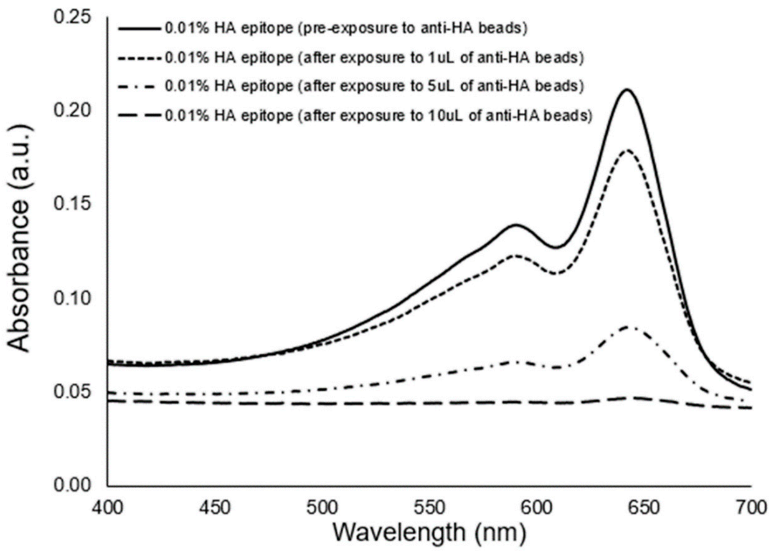

Figure S3. Absorption spectra of 0.01% HA epitope displaying vesicles showing comparison of clearance of vesicles as a function of anti-HA bead concentration.

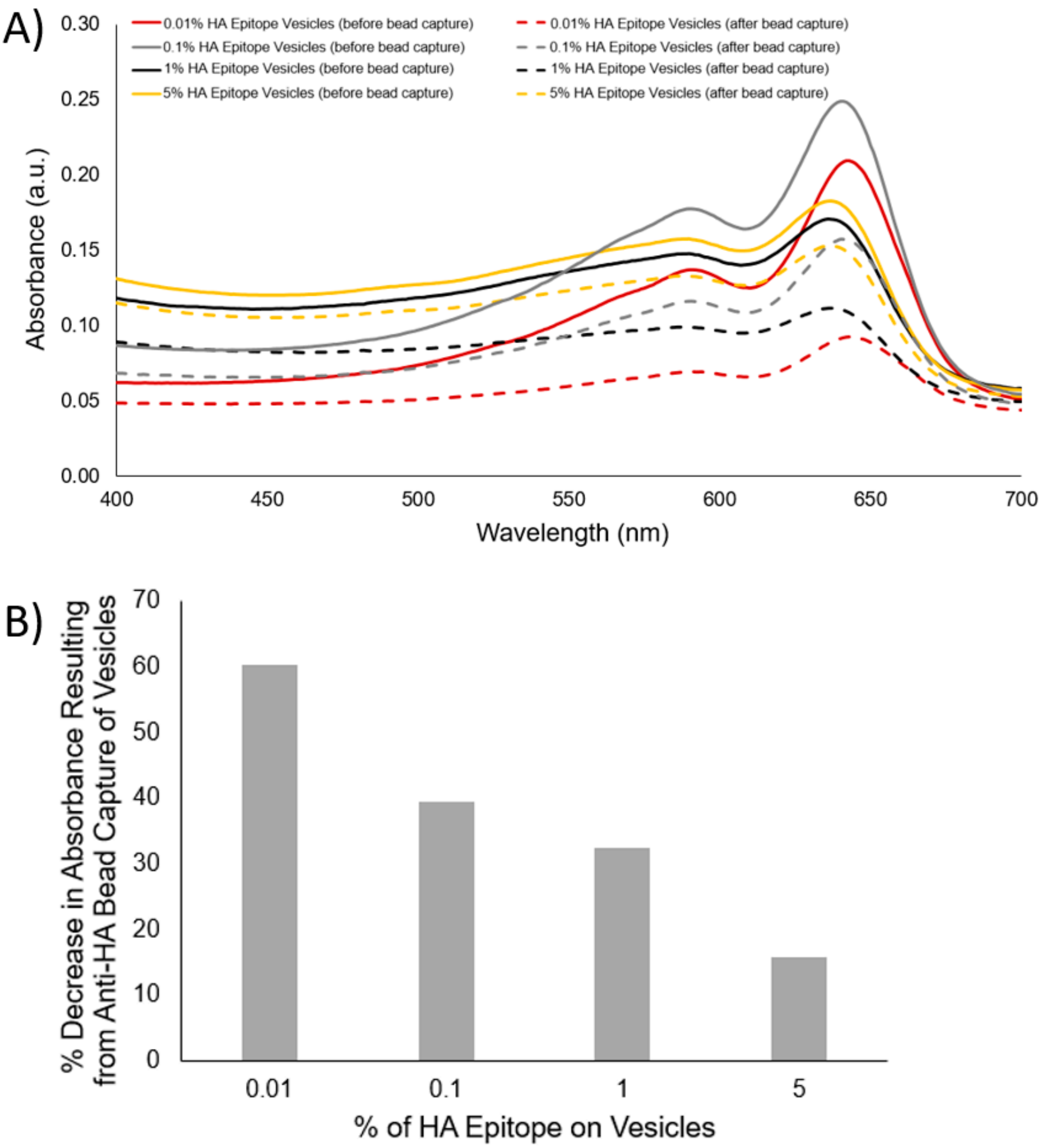

Figure S4. (**A**) Absorption spectra of vesicle suspension before and after exposure to 5uL of anti-HA beads for vesicles displaying different percentages of HA epitope. (**B**) Comparison of attenuation in signal resulting from vesicle capture by the anti-HA beads as a function of the different vesicle compositions (percentages of displayed HA epitope).
